# Diminution of pharyngeal segmentation and the evolution of the amniotes

**DOI:** 10.1186/s40851-019-0123-5

**Published:** 2019-02-11

**Authors:** Subathra Poopalasundaram, Jo Richardson, Annabelle Scott, Alex Donovan, Karen Liu, Anthony Graham

**Affiliations:** 1 Centre for Developmental Neurobiology, King’s College London, London, UK; 2 Centre for Craniofacial and Regenerative Biology, King’s College London, London, SE1 1UL UK

**Keywords:** Pharyngeal segmentation, Pharyngeal pouch, Pharyngeal arch, DLX, Amniote evolution, Larynx

## Abstract

**Background:**

The pharyngeal arches are a series of bulges found on the lateral surface of the head of vertebrate embryos, and it is within these segments that components of the later anatomy are laid down. In most vertebrates, the post-otic pharyngeal arches will form the branchial apparatus, while in amniotes these segments are believed to generate the larynx. It has been unclear how the development of these segments has been altered with the emergence of the amniotes.

**Results:**

In this study, we examined the development of pharyngeal arches in amniotes and show that the post-otic pharyngeal arches in this clade are greatly diminished. We find that the post-otic segments do not undergo myogenesis or skeletogenesis, but are remodelled before these processes occur. We also find that nested DLX expression, which is a feature of all the pharyngeal arches in anamniotes, is associated with the anterior segments but less so with the posterior arches in amniotes. We further show that the posterior arches of the mouse embryo fail to properly delineate, which demonstrates the lack of function of these posterior segments in later development.

**Conclusion:**

In amniotes, there has been a loss of the ancestral “branchial” developmental programme that is a general feature of gnathostomes; myogenesis and skeletogenesis This is likely to have facilitated the emergence of the larynx as a new structure not constrained by the segmental organisation of the posterior pharyngeal region.

## Background

The development of the pharyngeal arches is underpinned by the generation of the pharyngeal pouches, outpocketings of the endoderm, which contact the overlying ectoderm, with the other constituents of the arches, the neural crest and mesoderm, migrating into these preformed segmental units [1]. These embryonic populations differentiate to form a range of derivatives: the neural crest gives rise to the skeletal and connective tissues, the mesoderm to the musculature and the blood vessels, the ectoderm to the epidermis and sensory neurons and the endoderm the lining of the pharynx and a range of specialised organs. Thus, the arches constitute an iterated series with each forming the same components. In many vertebrate clades, this embryonic segmental organisation is translated into the later functional anatomy and is evident in the serial arrangement of the gill-bearing branchial arches.

In post-metamorphic amphibia and in amniotes, however, the branchial skeleton is lost and the larynx develops [2, 3]. This structure is a feature of tetrapods that connects the pharynx with the trachea and plays an essential role in facilitating life on land. The larynx consists of skeletal elements, which include the cricoid and paired arytenoids in most tetrapods as well as, in mammals, the thyroid, and associated muscular and connective tissue elements [2]. Developmentally, the components of the larynx are thought to have their origins in the posterior, post-otic, pharyngeal arches and thus to be equivalent to the derivatives of the posterior arches in other vertebrates, the branchial apparatus [2, 4]. Indeed, it is generally assumed that there is a correspondence between the mature structures of the larynx: skeleton, muscle and nerves, and the embryonic pharyngeal segments, and such a relationship is a staple of embryology and anatomy textbooks [4–9].

**Fig. 1:**
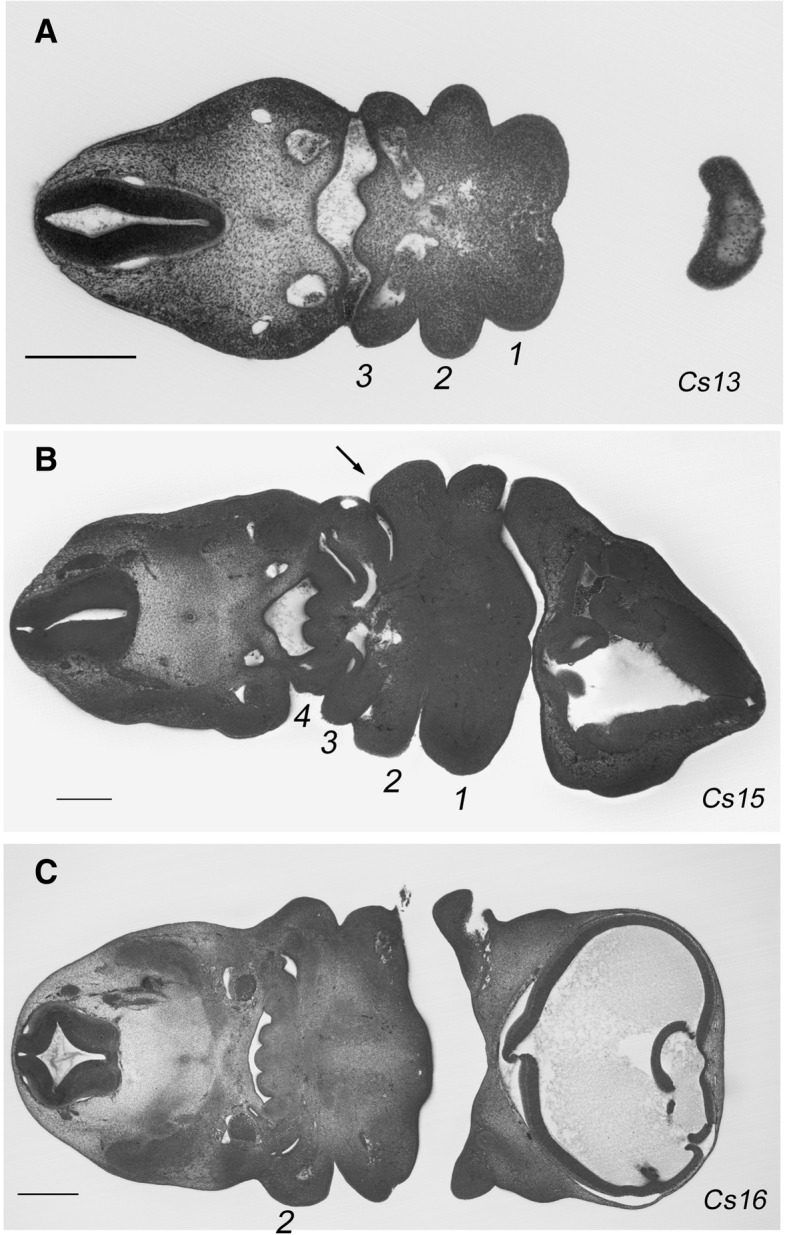
Segmental and post-segmental stages of pharyngeal development in human embryos. In this figure High-Resolution Episcopic Microscopy (HREM) data from the human embryos from the DMDD website (https://dmdd.org.uk/) is shown. **a** At Carnegie Stage (Cs) 13, the first three most anterior pharyngeal arches have formed and are morphologically evident. **b** By Cs 15 stage, a further fourth arch has formed. **c** However, by Cs16 the segmental nature of the pharyngeal region has been lost as the second arch has overgrown the posterior arches, covered and subsumed them. Scale bar = 0.25 mm

Yet, there have been few studies focussed on the development of the posterior arches and it is unclear whether such a correspondence exists or how the development of the posterior arches was modified to allow for the emergence of the larynx. Moreover, the situation in amniotes is more complex than that in other vertebrates, since the posterior pharynx is segmentally organised for only a transient period. Once all the arches have been formed, the second arch expands disproportionately to cover the posterior arches before fusing and enclosing them [10]. Figure 1 shows an overview of this process in human embryos. At Carnegie stage 13, the three most anterior arches have formed (Fig. 1a), while by stage 15 an additional fourth arch has formed (Fig. 1b). Thus, at these stages the segmental nature of the pharynx is readily apparent. However, by stage 16 the second arch has expanded caudally, covered and subsumed the posterior arches and thus the segmental nature of the pharyngeal region is lost (Fig. 1c). Consequently, the segmental nature of the posterior pharynx becomes lost.

In this study, we assessed the early development of the posterior pharyngeal arches and their subsequent envelopment by the second arch. We further document the relationship between the pharyngeal segments and the formation of the muscular and skeletal derivatives. We find that while there is a clear correspondence between these events and the anterior arches, this is not the case for the posterior segments. Thus, while the processes of myogenesis and chondrogenesis are underway at several sites in the embryo, they are not a feature of the posterior pharyngeal region. We further show that, although the anterior pharyngeal segments exhibit nested *DLX* gene expression, the expression of these genes is greatly reduced in the posterior arches, suggesting that the need to regionalise the neural crest along the proximodistal axis in these segments is less in amniotes. We note that the reduction in *DLX* expression is more extensive in the mouse than in the chick, and this prompted us to further investigate differences between the posterior segments in these species. Interestingly, we find that while the 4th pharyngeal pouch contacts the ectoderm in chick and human embryos, it does not do so in mice. Consequently, the posterior pharyngeal segments are not fully delineated in this species, which further underscores their lack of significance for later events.

## Results

**Fig. 2:**
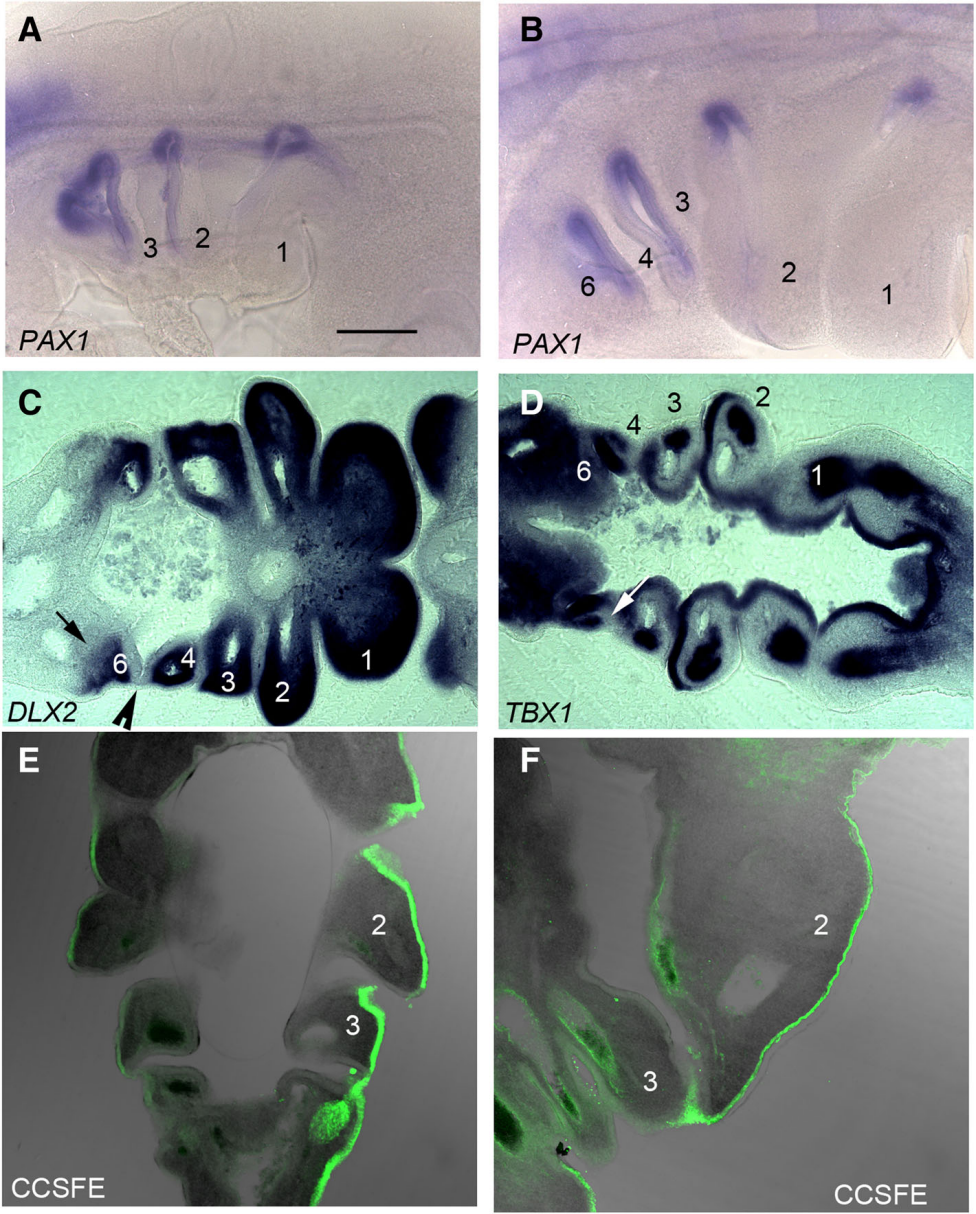
The generation and remodelling of the pharyngeal segments. **a** Side view of a HH17 chick embryo, the three fully formed anterior arches can be seen while at HH21 (**b**) the full complement of arches have formed. PAX1 highlights the position of the intervening pouches. (**c**) Longitudinal section of a HH21 chick embryo, DLX2 expression shows the neural crest component of the arches. Noticeably, the caudal edge of arch 6 has no clear definition; highlighted by the black arrow while the anterior limit of this arch is delineated by the pouch of contact between the fourth pouch and the overlying ectoderm, indicated by the black arrowhead. **d** Longitudinal section of a HH21 embryo, TBX1 expression demonstrates the relative positions of the mesoderm components of each of the arches as well as the pharyngeal endoderm and pouches. A sizeable mesodermal population can be seen in arches 1, 2, and 3. Arch 4 by comparison only has a reduced mesoderm component; indicated by the white arrow. At HH20 (**e**) the second arch is beginning to expand and by HH24 (**f**) it has overgrown the posterior arches. The arches are numbered in all panels. Scale bar = 0.1 mm

We conducted a detailed analysis of the period covering the formation of the post-otic pharyngeal segments and their subsequent envelopment by the second arch in chick. At stage 17 (HH17), *PAX1* staining highlights the formed, and forming, pharyngeal pouches and it is apparent that the first three arches are delineated, but that the more posterior segments are not clearly defined (Fig. 2a). By HH21, however, the full complement of four pouches and five arches—numbered 1,2,3,4 and 6—have formed (Fig. 2b). In amniotes, the most posterior pharyngeal arch is termed the sixth, even though this is numerically the fifth arch, due to the long held, but erroneous, belief that a transient fifth arch formed between this segment and the fourth [11]. We have also used *DLX2* and *TBX1* expression to highlight the distribution of the different embryonic populations that contribute to the arches. *DLX2* is expressed by the neural crest cells and it can be seen in all the arches (Fig. 2c). However, significantly, this staining highlights the fact that while the anterior of the 6th arch has a distinct anterior boundary, the point where the fourth pouch contacts the ectoderm, it does not have a defined caudal limit (Fig. 2c). *TBX1* expression labels the pharyngeal endoderm, including the pouches, as well as the mesodermal components of the first four arches (Fig. 2d). As development progresses the caudal edge of the second arch expands to cover the more posterior arches. This is shown in embryos in which the ectoderm has been labelled with a cell tracker, CCSFE. At HH20 the second arch is enlarging and that the point of expansion is at the interface between the ectoderm, which is labelled, and the endoderm (Fig. 2e). This process continues as the 2nd arch increases greatly in size and overhangs the posterior segments (Fig. 2f). Thus, in amniotes there is an earlier segmental phase of pharyngeal development and a later post-segmental phase.

**Fig. 3:**
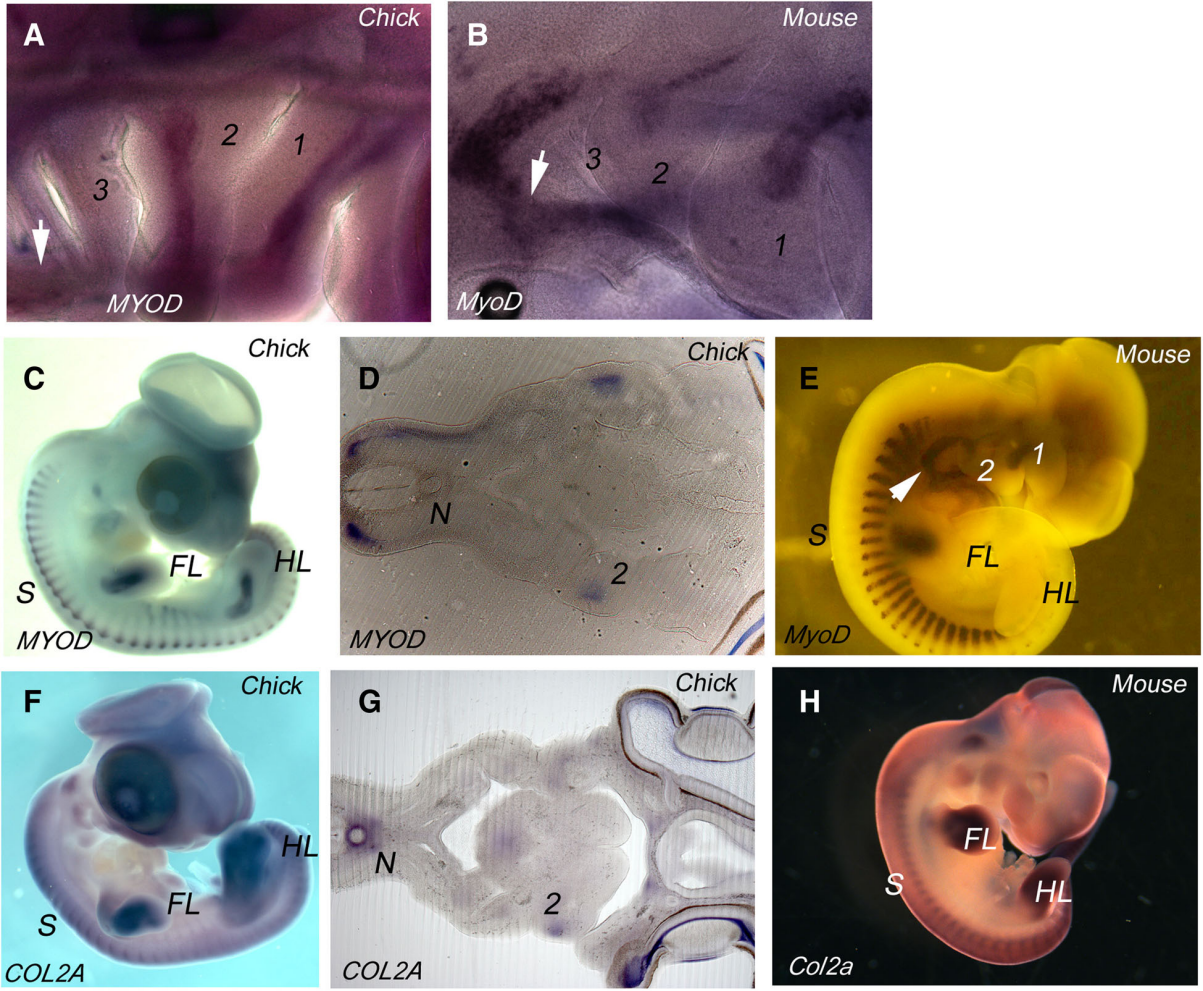
Myogenesis and chondrogenesis at segmental and post-segmental pharyngeal stages (**a**) *MYOD* expression in a HH21 chick embryo. Myogenesis can be seen to be occurring in the first two arches but not the more posterior arches. The migratory hypoglossal myoblasts, which are somite derived can be seen to migrate through the ventral pharyngeal midline region – white arrow. **b** *MyoD* expression in a TS16 mouse embryo. Myogenesis can be seen to be occurring in the first two arches, but not the more posterior arches. The somite derived hypoglossal myoblasts can be seen to be migrating around the caudal aspect of the pharyngeal arches and along the ventral pharyngeal midline, indicated by the white arrow. **c** *MYOD* expression in chick embryo at HH25. Ongoing myogenesis can be seen to be occurring in the somites and within the limb buds. There is some myogenesis apparent in the extended second pharyngeal arch, but not in the posterior pharynx. **d** Longitudinal section through the pharyngeal region of a HH25 chick embryo. *MYOD* expression within the second arch is apparent, but there is no expression in the more posterior pharyngeal region. *MYOD* expression is also seen in the somites. The position of the notochord (N) is marked. **e** *MyoD* expression in mouse TS17 embryo. Myogenesis is associated with the somites, developing limb buds and anterior pharyngeal arches, but is absent from the posterior pharyngeal region, except for expression in the hypoglossal myoblasts, indicated by the white arrow. **f** *COL2A* expression in HH25 chick embryo. Ongoing chondrogenesis can be seen to be occurring in the somites and limb buds. There is some chondrogenesis apparent in the extended second pharyngeal arch but not in the posterior pharynx. **g** Longitudinal section through the pharyngeal region of a chick embryo at HH25. *COL2A* expression within the second arch is apparent, as is expression around the notochord (N) but there is no expression in the more posterior pharyngeal region. **h** *Col2a* expression in mouse TS17 embryo. Chondrogenesis is associated with the somites, developing limb buds and anterior pharyngeal arches, but is absent from the posterior pharyngeal region

It has been widely documented that in anamniotes myogenesis and skeletogenesis occur within the segmental framework of the pharyngeal arches [12–14]. We therefore sought to determine if there is a relationship between muscular and skeletal differentiation and the segmental organisation of the arches in amniotes, and we used cell type specific markers on both chick and mouse embryos, as representatives of reptiles and mammals respectively. To analyse the emerging muscle of the arches, we used *MYOD* staining. In both HH21 chick and Theiler stage (TS) 16 mouse embryos, where the full cohort of arches have formed, we find that muscle differentiation is associated with arches 1 and 2, but not with the more posterior arches (Fig. 3a, b). However, other muscle populations are differentiating in the head, most notably the hypoglossal musculature which migrates from the occipital somites into the ventral pharyngeal region (Fig. 3a, b). We found no expression of *collagen IIA* (*COL2A*), which is a definitive cartilage marker, at any site in the pharyngeal arches at these segmental stages in either chick or mouse (data not shown).

We further analysed muscle and cartilage differentiation at later stages while the second arch is covering the posterior arches. In chick at HH25, *MYOD* staining can be seen in the first and second arch, but not in the more posterior pharynx (Fig. 3c, d), even though these segments have a resident mesodermal population (Fig. 2). However, it is also clear that myogenesis is well underway in other areas of the embryo including the myotome of the somites and myoblasts migrating into the limbs. Similarly, in the mouse at TS17, myogenesis is evident in the first two arches, in the myotome and in the myoblasts populating the forelimb. Yet, there is no myogenesis within the posterior arches at this stage, bar the migratory hypoglossal myoblasts (Fig. 3e). We also used *COL2A* staining to reveal sites of chondrogenesis at these later stages. We find that, while there is extensive chondrogenesis in the somites and the limbs, in the chick at HH25, there is little chondrogenesis in the pharynx. *COL2A* staining is evident in the first and second arches, but not in the more posterior arches (Fig. 3g). In the mouse at TS17, we also found chondrogenesis underway in the somites and limbs, and while there was some staining in the first two arches, the posterior was devoid of *COL2A* expression (Fig. 3h). Thus, we find that the posterior pharyngeal segments do not entertain myogenesis or skeletogenesis during their period of definition.

**Fig. 4:**
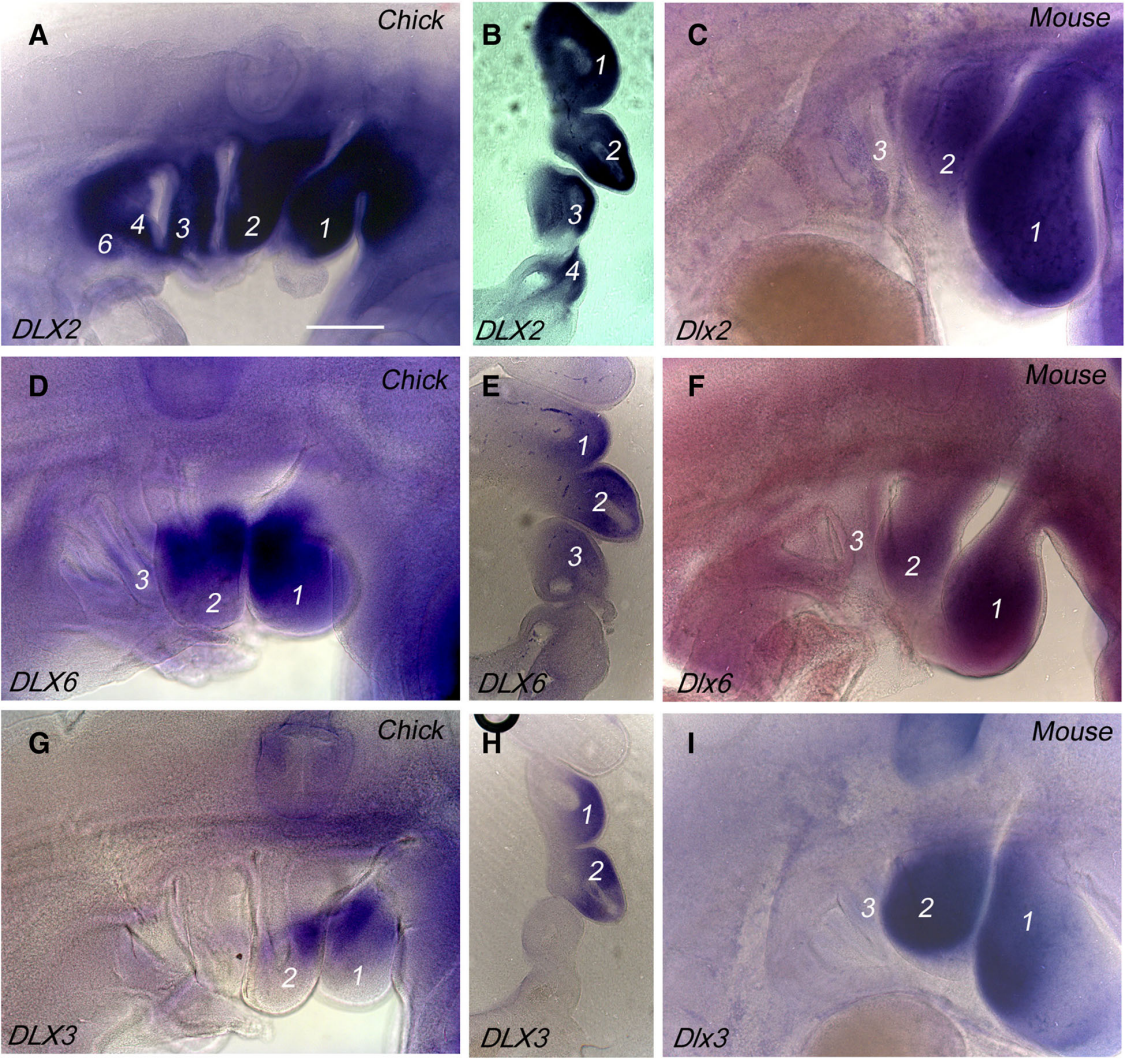
DLX expression in the pharyngeal arches in chick and mouse embryos. **a** Side view of a HH21 chick embryo showing *DLX2* expression throughout the dorsoventral extent of all the pharyngeal arches. **b** Longitudinal section through the arches showing *DLX2* expression in the ectomesenchyme of the arches. **c** Side view of a TS16 mouse embryo showing pronounced expression of *Dlx2* in the first two arches, but much reduced expression in the posterior arches. **d** Side view of a HH21 chick embryo showing *DLX6* expression in the mid region of the three most anterior arches but not the most posterior arches. **e** Longitudinal section through the arches showing *DLX6* expression in the ectomesenchyme of the three most anterior arches. **f** Side view of a TS16 mouse embryo showing high levels of *Dlx6* in the mid region of the first two arches, but much reduced expression in the posterior arches. **g** Side view of a HH21 chick embryo showing *DLX3* expression in the more distal region of the two most anterior arches but not those lying posteriorly. **h** Longitudinal section through the arches showing *DLX3* expression in the ectomesenchyme of the two most anterior arches. **f** Side view of a TS16 mouse embryo showing high levels of *Dlx3* in the distal region of the anterior two arches, but much reduced expression in the posterior arches. Scale bar = 0.1 mm

We further assessed the degree to which the pharyngeal arches are regionalised as they develop. The patterning of the neural crest and its skeletal derivatives along the proximodistal axis of the arches involves the nested expression of members of the *DLX* family of transcription factors. *DLX* genes are organised as linked pairs—*DLX1/2*, *DLX3/4* and *DLX5/6*—with *DLX 1/2* being expressed throughout the ectomesenchyme of the arches, *DLX 3/4* at the distal portion and *DLX 5/6* from the mid region distally. This situation is believed to be a general feature of gnathostomes [15–18]. Most studies of *DLX* expression in amniotes have focussed on the anterior arches and a directed study of the more posterior arches is lacking. We have therefore documented the expression of each of the linked *DLX* pairs in both chick and mouse embryos. In chick, we find that, as for other gnathostomes, *DLX2* is expressed at high levels throughout the ectomesenchyme all along the proximodistal axis of all the arches (Fig. 4a, b). However, the expression patterns of *DLX6* and *DLX3* differed from that seen in anamniotes. In chick, *DLX6* shows graded expression across the arches. This gene is strongly expressed in the mid region of the first two arches and there is some expression in the third arch, but we find less *DLX6* expression in the more posterior arches (Fig. 4d, e). *DLX3* shows a more restricted expression; again, this gene is strongly expressed in the first two arches but arches 3, 4 and 6 show little expression (Fig. 4g, h). The situation in mouse is similar in that *Dlx2*, *Dlx6* and *Dlx3* show nested expression in the first two arches, but in this species we find that for all these genes, including Dlx2, there is little expression in the posterior arches (Figu. 4c, f and i). Similar results have been found from other studies using alternative approaches with *Dlx1/2-cre/rosa 26* and *Dlx5–6 cre/rosa26* crosses shows lacZ labelling in the first two arches but not in the posterior arches [19, 20].

**Fig. 5:**
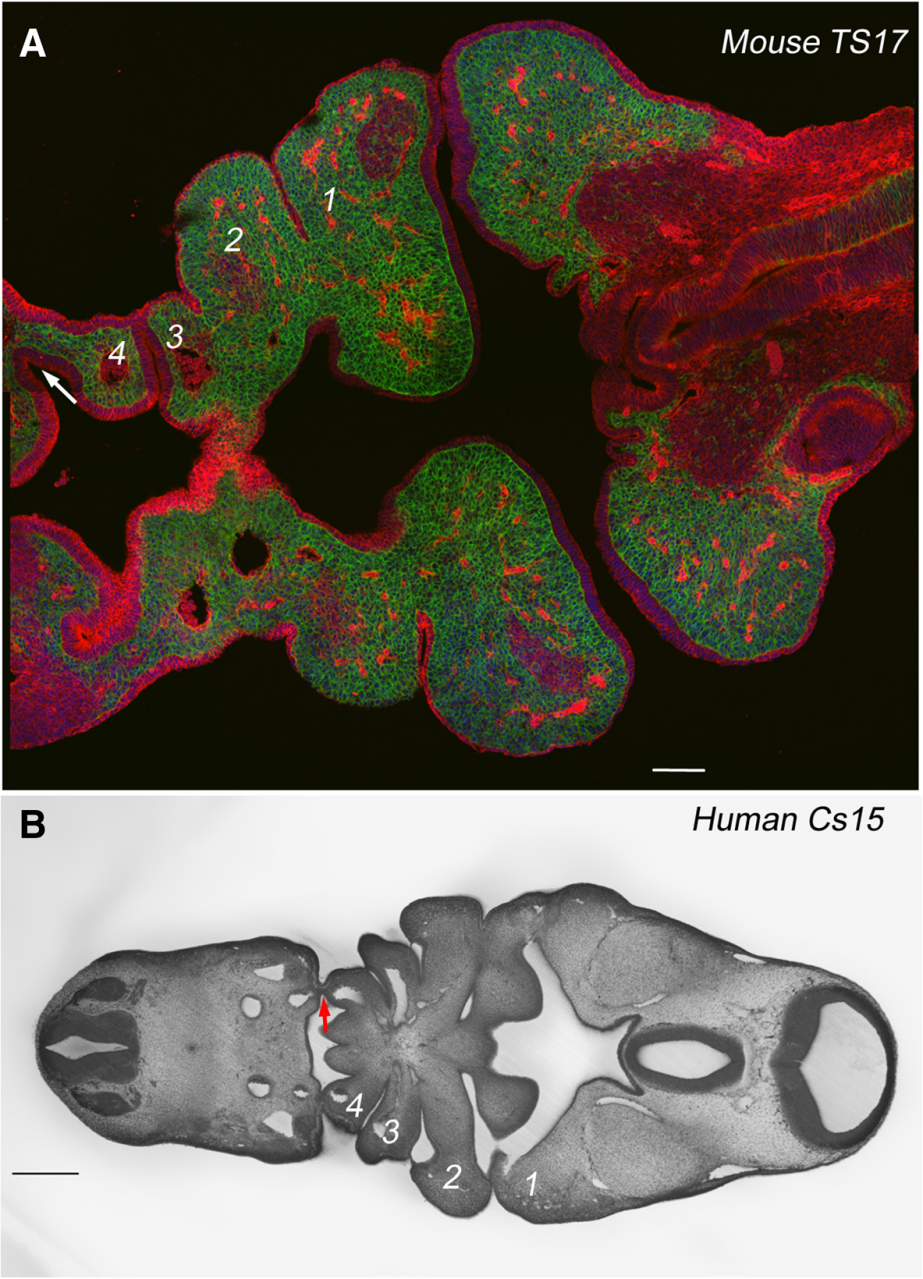
Pharyngeal pouch morphology in mouse and human embryos. **a** Confocal section through the pharyngeal region, at the segmental phase, of a TS17 (E10.5) mouse embryo. Neural crest (*Wnt1::cre+*) structures are labelled with membrane GFP, while non-neural crest tissues express membrane RFP. It is noticeable that the fourth pouch, highlighted by an arrow, does not contact the ectoderm. **b** HREM section through the pharyngeal region, at the segmental phase, of a CS15 human embryo. The fourth pouch, highlighted by an arrow, does contact the ectoderm. Scale bar = 0.25 mm

These differences in *DLX* expression in the posterior arches between chick and mouse prompted us to further analyse the underlying organisation of the arches. Central to the organisation of the pharyngeal arches is the establishment of the contact between the pharyngeal pouches and the overlying ectoderm. This serves to define the anterior and posterior limits of the arches and to segregate the mesenchymal populations of the different arches. We have previously documented the formation of the pharyngeal pouches and arches in detail in chick [21] and we therefore assessed if the topology of the mouse pouches and arches were the same. Notably, we find that in mouse the fourth pouch does not contact the ectoderm and thus neither establishes a clearly delineated posterior boundary of the fourth arch nor an anterior boundary of the sixth arch (Fig. 5a). We further analysed HREM (high-resolution episcopic microscopy) data of 25 E10.5 mouse embryos (TS16/17) catalogued on the DMDD website (https://dmdd.org.uk/), to determine if the failure of the fourth pouch to contact the ectoderm represented a temporal effect. However, we found that in every embryo analysed, the fourth pouch does not contact the ectoderm and this was observed in embryos that varied between 3.7 mm to 5.2 mm crown rump length, and that were either in the segmental or early post-segmental phases of development. We further analysed human embryo resources to determine if this is a general feature of mammals or a derived feature of mice. We examined HREM data from the four Carnegie stage (CS)15 human embryos catalogued on the DMDD website; this is the stage in humans at which all the arches have formed. In each we noted that the fourth pouch could be seen to contact the ectoderm (Fig. 5b). This same topology was also found in histological sections of a CS15 embryo archived on the Virtual Human embryo database (https://www.prenatalorigins.org/virtual-human-embryo/stage.php?stage=15). Thus, the mouse represents a test of whether the failure to fully establish the posterior pharyngeal segments has any impact on later anatomy; clearly, it does not, and the larynx forms.

## Conclusions

A key observation from this study is that the development of the posterior pharyngeal arches in amniotes is markedly different from that of other vertebrate clades. In anamniotes, the posterior arches generate muscular and skeletal structures, exhibit nested expression of *DLX* linked pairs and these segments form the blueprint for the organisation of the branchial apparatus [12, 15–18, 22]. We show here that in chick and mouse the posterior arches do not undergo myogenesis or skeletogenesis while they exist as morphologically discernible entities, and that they exhibit reduced nested expression of the *DLX* genes; there has been a suppression of the inner fish here. Consequently, the segmental organisation of the posterior embryonic pharynx has little significance for the later musculoskeletal anatomy; a point underscored by the fact that, in mouse, the posterior arches fail to fully emerge. Thus, these results lay bare the fact that the correspondence between the pharyngeal arches and the components of the larynx, invariably found in anatomy and embryology textbooks, is but supposition [4, 6–9].

Finally, it should also be noted that while the skeletal components of the branchial apparatus of anamniotes are generally neural crest derived [23, 24], this is not the case for the larynx. Fate mapping studies in birds have demonstrated that the laryngeal cartilages, the cricoid and arytenoid, arise from the lateral plate mesoderm [25]. Similarly, a recent study in mouse has also shown a mesodermal origin for the cricoid and arytenoid cartilages [26]. However, the thyroid cartilage, which is a mammalian novelty, seems to have a more complex origin comprising both a crest derived and a mesodermal component [26]. Thus, the evolution of the larynx cannot have involved a transformation of the developmental programme that would have directed the generation of the branchial apparatus, but rather the larynx emerges as a new structure.

## Materials and methods

### Embryo staging

Chick embryos were staged according to Hamburger and Hamilton [27] and mice according to Theiler [28]. The following mouse lines were used: Wnt1-cre driver: Tg (Wnt1-cre)11Rth [29] and reporter line: R26RmT/mG: GT (Rosa)26Sortm4(ACTB-tdTomato-EGFP) [30]. All animal work was performed in accordance with UK Home Office Regulations. The HREM data from the human embryos is from the DMDD website (https://dmdd.org.uk/). Data from Deciphering the Mechanisms of Developmental Disorders (https://dmdd.org.uk/), a programme funded by the Wellcome Trust with support from the Francis Crick Institute, is licensed under a Creative Commons Attribution licence.

### In situ hybridisation

Embryos were fixed in in 3.7% (*v*/v) formaldehyde in PBS and in situ hybridisation carried out as previously described [31]. Gene expression was either examined as whole mounts or embryos were embedded in 20% (*w*/*v*) gelatin in PBS and sectioned using a vibratome.

### CCSFE labelling

CCSFE labelling was carried out as previously described [10].
